# Emotion recognition and false belief in deaf or hard-of-hearing preschool children

**DOI:** 10.1093/deafed/enad044

**Published:** 2023-10-07

**Authors:** Emrah Akkaya, Murat Doğan

**Affiliations:** Education of Hearing Impaired, Department of Special Education, Faculty of Education, Anadolu University, Eskişehir, Turkey; Education of Hearing Impaired, Department of Special Education, Faculty of Education, Anadolu University, Eskişehir, Turkey

## Abstract

This study aims to examine emotion recognition and false belief performances of 4–5-year-old (48–71 months) deaf or hard-of-hearing (DHH) children. The performances have been assessed using the Turkish Version of the Theory of Mind Task Battery for Children. The DHH children have been continuing schooling in inclusive settings with an auditory-oral approach. The emotion recognition performances of hearing children (*n* = 100) and DHH (*n* = 100) children have appeared to be similar. The ANOVA analysis has revealed that the groups do not differ concerning false belief performances between the ages of 4 and 5.5. However, from the age of 5.5, hearing children have performed better than DHH children. According to correlation analysis, parental education has been determined as a remarkable factor in DHH children’s false belief development. The findings point to the need for research across a wide range of ages to better understand the developmental course of false belief in DHH children.

## Introduction

People use theory of mind (ToM) to interpret many mental states such as understanding emotions, desires, beliefs, and jokes, distinguishing lies, and recognizing tricks. Having a ToM or a representational grasp of how ideas and emotions influence behavior is essential for social interaction and reasoning (Peterson & Wellman, 2019). As social beings, humans can make inferences about the mental states of others by taking their own mental state and the other person’s behavior as reference points. Thus, the ToM is a social–cognitive process that involves the ability to understand one’s own point of view and those of others as well as to grasp the relationship between the mind and action (Leslie et al., 2004). In this context, ToM is defined as the ability to understand, distinguish, and refer to one’s own and others’ mental states such as emotions, intentions, desires, and beliefs (Cole, 2006; Frith & Frith, 2005; Penn et al., 2006).

The literature is densely populated with studies conducted on ToM for nearly 40 years. At first, ToM research focused on false belief understanding and subsequently on various dimensions in the development of ToM, such as intention, emotion, desire, knowledge access, false belief, irony, and the understanding of faux pas (O'Reilly et al., 2014; Peterson & Wellman, 2019; Recchia et al., 2010; Wellman & Liu, 2004). Wellman and Liu have suggested a generally accepted developmental order of ToM, which are diverse desires, diverse beliefs, knowledge access, false belief, and hidden emotion. This order is age-related and may vary according to the culture in which children live (Shahaeian et al., 2011). It is argued that the developmental sequence by Wellman and Liu continues with the advanced ToM understanding at school age and beyond. Accordingly, the second and higher order of false belief, irony, faux pass, jokes, and white lies are conceptualized as the advanced ToM (Miller, 2009; Osterhaus et al., 2016).

### Emotion recognition and false belief in ToM

An important developmental milestone in early childhood is reached when a child better understands her/his own emotions and those of others (Carpendale & Lewis, 2010). During this period, young children increasingly understand that one’s mental state can evoke certain emotions, that facial expressions reflect emotions, and that emotions influence behavior, and can be used to influence the emotions of others (Cole et al., 2009). By the age of 2, children can understand that their own desires and emotions may be different from those of other people, and they can start talking about these desires and emotions (Wellman, 2014). This development in emotion recognition forms the basis for understanding hidden emotions, emotions linked to desires, and false beliefs (Saxe & Houlihan, 2017; Ziv et al., 2013). Accordingly, emotion recognition is a prerequisite skill for understanding desires and false beliefs (Wang et al., 2014). To summarize, since understanding diverse desires, diverse beliefs, hidden emotions, and false beliefs are among the components of ToM, it is important to acquire emotion recognition skills to understand emotions related to these components.

False belief understanding means that an individual observing the event from the outside predicts the behavior or thoughts of the people involved in the event according to the situation they are in (Call & Tomasello, 2008). This prediction allows the attribution of beliefs to those people about the event. For example, Little Red Riding Hood believes that her grandmother is waiting for her at home whereas children know that the wolf is in her grandmother’s bed. Although false belief understanding may seem complex, children begin to understand false beliefs, which is central to ToM, at age 4 (Wellman, 2014) and can complete false belief tests at age 5 (Wellman et al., 2011). These ages can be considered periods in which the developmental speed increases and the differences emerge in terms of ToM. For this reason, both the initial and current studies on ToM generally have focused on false belief understanding.

### Emotion recognition in deaf or hard-of-hearing children

Although it has been reported that deaf or hard-of-hearing (DHH) children experience a delay in ToM development (Holmer et al., 2016; Marchetti et al., 2006; Meristo et al., 2012; Netten et al., 2017; O'Reilly et al., 2014; Peterson et al., 2016; Peterson & Wellman, 2019; Walker et al., 2017), studies on emotion recognition in DHH children have revealed contradictory results. For instance, while Tsou et al. (2021) stated that the emotion recognition performances of DHH children between the ages of 3 and 10 were similar to those of their hearing peers, Gray et al. (2007) reported that 7–11-year-old DHH children underperformed compared to their hearing peers in matching the characters’ emotions with the picture of their facial expressions in illustrated scenarios.

Hao and Su (2014) concluded that DHH children between the ages of 9 and 13 made use of clear visual cues in facial and bodily expressions in their emotion recognition skills. Despite the cues in the visual expression, Dyck et al. (2004) determined that 6–11 and 12–18-year-old DHH children performed poorer on emotion recognition tasks than children with visual impairments and hearing children. However, when the language variable was controlled, they found that DHH children performed as well as or better than the others.

Most and Michaelis (2012) stated that DHH children between the ages of 4 and 5 performed poorer in emotion recognition tasks compared with hearing children even though the tasks were presented in auditory, visual, and audio-visual forms. In line with this research, Huttunen and Ryder (2012) reported that 3–7-year-old DHH children started to differ from hearing children in the use of emotional expressions as of 4 years of age. However, Ziv et al. (2013) stated that DHH and hearing children aged between 5 and 7 had comparable performances in emotion recognition tasks derived from the context and facial expressions. In summary, the performances of DHH children in emotion recognition varied even though they were similar in age to hearing children and the tasks were presented in various forms.

### False belief in DHH children

In the first study examining the false belief skills of DHH children, Peterson and Siegal (1995) stated that 65% of 8–13-year-old DHH children performed poorly on the false belief task, and only half of them were successful even when the tasks were presented interactively. In subsequent research, false belief performances of DHH children using sign language from birth were found similar to those of hearing children. It was determined that DHH children using sign language from birth outperformed DHH children who used oral language and had hearing parents (Courtin & Melot, 2005; Meristo et al., 2007; Meristo & Hjelmquist, 2009; Schick et al., 2007; Wellman et al., 2011). However, some studies have reported several similarities (Pluta et al., 2021; Ziv et al., 2013) and differences (Remmel & Peters, 2008; Stanzione, 2014) between the false belief performances of DHH children with cochlear implants and those of their hearing peers.

Recent studies on DHH children’s performance in terms of false belief understanding have concluded varying results. In a 2-year longitudinal study, Smogorzewska and Osterhaus (2021) examined second-order false belief understanding in children between the ages of 7.5 and 9.5 (*N* = 779). They reported that hearing children performed better than DHH children on second-order false belief measures. They also stated that even though DHH children grew older, the performance gap between them and their hearing peers did not close. However, in another longitudinal study, Walker et al. (2017) examined the false belief performances of 5–6-year-old and second-grade level DHH children. While the false belief performances of hearing children were better than those of DHH children at a young age, they noted that there was no difference between the groups at the second-grade level.

### Present study

In Turkey, the education of DHH children during the preschool period (36–72 months) is provided by special education teachers and preschool teachers in special education kindergartens or in general preschool education institutions that have inclusive settings. In addition to this education, DHH children benefit from related services in their schools and special education rehabilitation centers (Ministry of National Education, 2021). The auditory-oral approach is adopted in public schools and special education rehabilitation centers (Tüfekçioğlu, 2010). Although there is no sign language-based education in Turkey, some DHH individuals use Turkish Sign Language in daily life (Acar et al., 2020), which was accepted as the Turkish Sign Language by the Turkish Grand National Assembly in 2005. There are ongoing linguistic studies for the development of Turkish Sign Language (Acar et al., 2020; Dikyuva et al., 2015; Kubuş et al., 2016).

In Turkey, only a few studies have examined ToM skills in DHH children. In the first study, DHH children aged 4–7 years (*n* = 12) performed poorer in false belief tasks than hearing children aged 3–8 years (*n* = 17), and it was reported that the false belief performances of DHH children were not correlated with mother–child communication (Sancar, 2004). In another study (Sarıkardaşoğlu, 2010) examining the relationship between 6- and 11-year-old DHH children’s level of understanding in terms of social concepts related to intention, emotion, and morality and their aggressive behavior, the author concluded that DHH children who could not understand the intentions of others showed problem behaviors to a greater extent. Kirazlı (2014) investigated the relationship between the false belief skills of 9–11-year-old children with cochlear implants and their general and emotional intelligence, facial expression and emotion recognition, and adaptation skills. Kirazlı reported that children who performed poorly in false belief tasks also underperformed in other skills. Alaylı (2015) concluded that receptive language and self-regulation skills were important in the development of ToM in DHH children between the ages of 3 and 12. The researcher also reported that the developmental order of the ToM was consistent with the developmental order addressed in the research by Peterson and Wellman (2019).

In studies on the false belief skills of DHH children in Turkey, the age ranges of the participants are wide, and most of them are older. In addition, only one study has examined the false belief skills of DHH children in the context of normal development, but the number of participants in that study was limited (Sancar, 2004). In the current research, there are more participants than in all these studies, and besides, the groups are matched by age and gender. It is also expected that this research will contribute to a greater understanding of ToM in DHH children beyond the Western world.

ToM emerges gradually, beginning with the emergence of intuitive social skills in infancy and progressing toward the development of social cognition in toddlers and preschoolers. Around the age of 4 or 5, children begin to realize that people can have different opinions, that people can sometimes have false beliefs, and that this can affect their actions or words (Astington & Edward, 2010). By the age of 2, children have already gained competence in emotion recognition skills (Carpendale & Lewis, 2010; Wellman, 2014). The acquisition of emotion recognition skills is important to understand and interpret the emotional consequences of false beliefs (Wang et al., 2014). According to the results, DHH children show different performances in emotion recognition and false belief understanding. Since emotion recognition skills are precursors for false belief understanding and false belief understanding is a skill that can be monitored at the age of 4–5, the current research was designed to examine the development of these skills in 4–5-year-old DHH children.

The research conducted on DHH children in Turkey regarding their emotion recognition and false belief skills has the potential to make a significant contribution to ToM literature. This research is expected to open new opportunities for cross-cultural studies. Moreover, equipping educators working in this field with knowledge about DHH children’s emotion recognition and false belief skills can guide them in planning their work more effectively.

The main purpose of this study is to examine the emotion recognition and false belief performances of DHH children from a developmental perspective. In addition, the study examines the relationships between emotion recognition, false belief skills, and variables specific to DHH children, such as age at the onset of hearing aid use, age at the onset of cochlear implant use, and age at diagnosis.

## Method

### Participants

The sample (*N* = 200) consisted of 4–5-year-old (48–71 months) DHH (*n* = 100) and hearing (*n* = 100) children attending preschool education. The characteristics of the participants are presented in Table 1.

**Table 1:** Characteristics of the sample (*N* = 200).

**Categorical variables**	**DHH (*n* = 100)**		**H (*n* = 100)**	
	***n***		***n***	
Gender				
Female	50		50	
Male	50		50	
Mothers’/fathers’ education				
Elementary education	54/37		28/16	
Secondary education	29/43		32/42	
Higher education	17/20		40/42	
Degree of hearing loss				
Moderate (41–70 dBHL)	27			
Severe (71–95 dBHL)	33			
Profound (≥96 dBHL)	40			
Hearing technology				
Hearing aid	33			
Cochlear implant	68			
**Continuous variables**	***n***	***M (SD)***	***n***	***M (SD)***
Age				
Group 1 (48–53 months)	26	50.0 (1.90)	24	50.9 (1.84)
Group 2 (54–59 months)	24	56.1 (1.79)	26	56.4 (1.75)
Group 3 (60–65 months)	23	62.8 (1.83)	31	62.1 (2.06)
Group 4 (66–71 months)	27	68.8 (1.44)	19	68.1 (1.59)
Age at diagnosis				
≤3 months	38	1.45 (.76)		
>3 months	62	13.6 (9.93)		
Age at the onset of hearing aid use				
≤6 months	37	4.86 (1.23)		
>6 months	63	17.7 (11.13)		
Age at the onset of cochlear implant use				
≤36 months	54	21.2 (6.59)		
>36 months	14	46.0 (5.92)		
Duration of mothers’ education (years)	100	8.84 (4.23)	100	12.0 (4.50)
Duration of fathers’ education (years)	100	9.96 (3.41)	100	13.0 (3.81)

*Note.* dBHL = decibel hearing loss; DHH = deaf or hard-of-hearing children; H = hearing children.

The DHH children in this study were preschoolers attending educational institutions located in seven provinces of Turkey (İstanbul, İzmir, Bursa, Kütahya, Ordu, Konya, and Eskişehir). All DHH children were taught oral language at school. In addition, most of these children had severe to profound hearing loss, had early cochlear implantation, and came from families where oral language was used intensively. One-third of the children had been diagnosed at a very early age and had started using hearing aids. The inclusion criteria for the DHH children were: (1) normal cognitive function according to the medical report, teacher report, and researcher observation, (2) no additional disability, and (3) using cochlear implant(s) and/or hearing aid(s). Four children with additional disabilities and two children with brainstem implants who were reported to have additional disabilities were not included in the study.

**Table 2:** Descriptive statistics by age, mothers’, and fathers’ education levels.

**Variables**	**Groups**		**N**	***M***	***SD***	**Min.**	**Max.**
Emotion recognition							
Age	DHH	Group 1 (48–53 months)	26	4.31	.679	3	5
		Group 2 (54–59 months)	24	4.38	.711	3	5
		Group 3 (60–65 months)	23	4.48	.593	3	5
		Group 4 (66–71 months)	27	4.70	.542	3	5
	H	Group 1 (48–53 months)	24	4.33	.482	4	5
		Group 2 (54–59 months)	26	4.65	.485	4	5
		Group 3 (60–65 months)	31	4.71	.461	4	5
		Group 4 (66–71 months)	19	4.68	.478	4	5
Mothers’ education	DHH	Elementary education	54	4.46	.636	3	5
		Secondary education	29	4.34	.670	3	5
		Higher education	17	4.71	.588	3	5
	H	Elementary education	28	4.46	.508	4	5
		Secondary education	32	4.63	.492	4	5
		Higher education	40	4.68	.474	4	5
Fathers’ education	DHH	Elementary education	37	4.38	.758	3	5
		Secondary education	43	4.47	.592	3	5
		Higher education	20	4.65	.489	4	5
	H	Elementary education	16	4.50	.516	4	5
		Secondary education	42	4.50	.506	4	5
		Higher education	42	4.74	.445	4	5
False belief							
Age	DHH	Group 1 (48–53 ay)	26	1.31	.838	0	3
		Group 2 (54–59 ay)	24	1.38	.924	0	3
		Group 3 (60–65 ay)	23	1.35	1.03	0	3
		Group 4 (66–71 ay)	27	1.41	1.05	0	3
	H	Group 1 (48–53 ay)	24	1.58	1.35	0	4
		Group 2 (54–59 ay)	26	2.12	1.34	0	6
		Group 3 (60–65 ay)	31	2.06	1.61	0	6
		Group 4 (66–71 ay)	19	3.74	2.16	0	7
Mothers’ education	DHH	Elementary education	54	1.20	.877	0	3
		Secondary education	29	1.38	.979	0	3
		Higher education	17	1.82	1.02	0	3
	H	Elementary education	28	1.75	1.67	0	7
		Secondary education	32	2.19	1.40	0	7
		Higher education	40	2.73	1.96	0	6
Fathers’ education	DHH	Elementary education	37	1.11	.875	0	3
		Secondary education	43	1.37	1.00	0	3
		Higher education	20	1.80	.834	0	3
	H	Elementary education	16	2.06	1.65	0	7
		Secondary education	42	1.86	1.51	0	6
		Higher education	42	2.79	1.91	0	7

*Note.* DHH = deaf or hard-of-hearing children; H = hearing children.

All hearing children were preschoolers at educational institutions in Eskişehir. To support the representational strength of this group, hearing children attending schools in different districts with different socioeconomic levels of Eskişehir were included in the study. The inclusion criteria for hearing children were: (1) normal cognitive functioning based on teacher report and practitioner observation, and (2) no disability. Four of these children were not included in the study because they had significant learning problems according to the teachers’ reports.

The groups were matched in terms of age and gender to minimize the influence of other factors on the test scores other than hearing status. For the detailed analysis of the development of false belief, DHH and hearing children’s groups were further subdivided into four groups with 6-month intervals (see Table 1). There was no significant difference between the mean ages of the groups at the same age interval (*t* = −1.73, *p* = .090 for Group 1; *t* = −.745, *p* = .460 for Group 2; *t* = −.215, *p* = .830 for Group 3; and *t* = −.806, *p* = .425 for Group 4). On the other hand, there was a significant difference between the duration of mothers’ education (*t* = −4.10, *p* = .000) and the duration of fathers’ education (*t* = −4.29, *p* = .000). Accordingly, the duration of mothers’ and fathers’ education was determined to be longer for the hearing children (see Table 1).

### Instruments

In the study, a participant information form and the Theory of Mind Task Battery for Children-Turkish Version (ToMTBC-TV) were used to collect information about the participants and to evaluate ToM skills including emotion recognition and false belief, respectively.

#### Participant information form

The participant information form consisted of 14 items for DHH children and 9 items for hearing children. Both forms contained items regarding demographic information while the DHH children’s form also included five additional items about hearing loss (degree of hearing loss, current hearing technology in use, age at diagnosis, age at the onset of hearing aid use, age at the onset of cochlear implant use). An informed consent form and the participant information form were presented together to the parents, and those who gave consent for participation were asked to complete the forms.

#### The Theory of Mind Task Battery for Children-Turkish Version

The original battery was developed by Hutchins et al. (2008), and it was adapted to Turkish by Altıntaş (2014). There is a total of 26 items in the test, 15 of which are for ToM skills, and 11 of them are control items to decide whether a participant can move on to the next stage of the test. In the adaptation study, the test was applied to hearing children and children with autism between the ages of 4 and 5. As a result of the test/re-test method, the stability coefficient of the test was reported as .95, the internal consistency coefficient as .84, and the total reliability as .73–.95. For the current research, the Cronbach α coefficient was .90.

The ToMTBC-TV is a battery applied to children individually. The events in the test, depicted in color and developed to evaluate ToM skills from simple to complex, are presented to the child through oral expression, and the relevant items devised for control or ToM are addressed. When a control item is answered correctly, the participant moves on to the next ToM item, and if it is answered incorrectly, the test is discontinued. Each correct and incorrect answer is scored with 1 and 0 points, respectively. The minimum score to be obtained from the test is 0, and the maximum score is 15. The ToMTBC-TV had been applied to hearing children and children with autism in previous use. It was applied to DHH children in Turkey for the first time in this research. Since the test does not have a structure that requires the participant to answer orally, the participant can answer the items by pointing to some visuals. Written explanations under the images about the relevant situation and the story-like order of the images make it easier to understand the item (Hutchins et al., 2008). Overall, the test was deemed appropriate for the assessment of ToM development in DHH children.

### Procedures

The research proposal (protocol) and informed consent forms were accepted and approved by the Board of Social and Human Sciences Scientific Research and Publication Ethics of Anadolu University and the Ministry of National Education.

A pilot study was carried out by the first author with 20 DHH and hearing children accommodated in accordance with the selection criteria of the research. During the pilot study, it was noted that neither group of children understood the items on perspective-taking. Therefore, the items on perspective-taking were excluded during actual testing considering that they would cause measurement errors affecting the reliability of the study and that the inability of these items to measure the perspective-taking skill would negatively affect validity. Only the emotion recognition and false belief items were employed in the testing phase. Items 1, 2, 3, 4, and 7 were used for the emotion recognition test, and items 8, 9, 10, 11, 12, 13, 14, and 15 were used for the false belief test along with the control items. The minimum–maximum scores were 0 to 5 in the emotion recognition test and 0 to 7 in the false belief test. The administration of the test required 6–17 min, and the data were collected for 3 months. Each child visited the center or school with a family member—typically the mother—to be administered the test. While the family member filled out the participant information form, the first author chatted socially with the child to establish rapport. DHH children’s tests were administered individually in special education rehabilitation centers’ classrooms, and hearing children’s tests were administered individually in their own schools in a stimuli-free setting by the first author. The first author holds both a bachelor’s and a master’s degree in the field of education of DHH individuals. He has 17 years of experience in this field and is now continuing his doctoral studies. He has been working on cognitive development under the supervision of an associate professor of psychology for 8 years.

### Data analysis

The SPSS for Windows version 25.0 was used in all statistical analyses. An independent samples *t*-test was performed to examine the emotion recognition and false belief scores according to the hearing status, diagnostic age of DHH children, age at the onset of hearing aid use, and age at the onset of cochlear implant use. The emotion recognition and false belief scores according to age and the mothers’ and fathers’ education levels were analyzed together with the hearing status using a two-way ANOVA. In two-way ANOVA, the mothers’ and fathers’ education levels were used as categorical variables, and in correlation analyses, the durations of the mothers’ and fathers’ education were used as continuous variables. The statistical significance value was accepted as *p* < .05. The exact value of *p* is reported in the results section.

## Results

The results are presented in three parts for convenience. Under the first heading, analyzes of DHH and hearing children’s emotion recognition and false belief scores according to hearing status, and analyzes of false belief scores according to hearing status–age; hearing status–mother’s education level; and hearing status–father’s education level are presented. The second heading includes comparisons of DHH children’s emotion recognition and false belief scores according to those variables specific to hearing loss (hearing technology in use, age at diagnosis, age at onset of hearing aid use, age at onset of cochlear implant use). Under the last heading, the correlation analyzes of DHH children’s emotion recognition and false belief scores with the continuous variables specific to DHH children are presented.

### Hearing status, age, and parental education

The independent samples *t*-test results demonstrated that DHH (*M* = 4.47, *SD* = .643) and hearing children’s emotion recognition scores (*M* = 4.60; *SD* = .49) did not differ significantly between the groups (*t* = −1.61, *p* = .110, ${\mathrm{\eta}}^2$ = .23). However, hearing children’s (*M* = 2.28; *SD* = 1.75) false belief scores were significantly higher (*t* = −4.63, *p* = .000, ${\mathrm{\eta}}^2$= .66) than those of DHH children (*M* = 1.36; *SD* = .948).

The descriptive statistics of the groups’ emotion recognition and false belief scores are presented in Table 2, and the ANOVA results are presented in Table 3. Since there was no significant difference in the emotion recognition scores according to hearing status, only false belief scores were analyzed.

**Table 3:** Examination of false belief scores according to age and mothers’ and fathers’ education levels with hearing status.

**Sources**	***SS***	***df***	***MS***	***F***	***p***	**Partial** ${\boldsymbol{\mathrm{\eta}}}^{\mathbf{2}}$	**Observed power**
Hearing status	50.7	1	50.7	28.9	.000^*^^*^^*^	.13	1.00
Age groups	33.1	3	11.0	6.28	.000^*^^*^^*^	.09	.963
Hearing status *×* age groups	28.1	3	9.38	5.34	.001^*^^*^^*^	.08	.929
Error	337	192	1.76				
Total	1.096	200					
Hearing status	25.0	1	25.0	13.1	.000^*^^*^^*^	.06	.949
Mothers’ education	18.4	2	9.21	4.83	.009^*^^*^	.05	.794
Hearing status *×* mothers’ education	1.07	2	.533	.279	.757	.00	.094
Error	370	194	1.91				
Total	1.096	200	9.21				
Hearing status	28.0	1	28.0	14.8	.000^*^^*^^*^	.07	.969
Fathers’ education	18.1	2	9.07	4.81	.009^*^^*^	.05	.792
Hearing status *×* fathers’ education	2.71	2	1.36	.718	.489	.01	.170
Error	365	194	1.89				
Total	1.096	200					

^*^^*^ *p* < .01, ^*^^*^^*^p < .001.

According to the two-way ANOVA results, the interaction effect of hearing status and age in false belief scores was significant (see Table 3). The main effect of age on false belief scores was also significant. In pairwise comparisons for all participants (*N* = 200), the false belief scores of children in Group 4 (66–71 months children) (*M* = 2.37, *SD* = 1.96) were higher than those of the children in Group 3 (60–65 months children) (*M* = 1.76, *SD* = 1.43, *p* = .009), Group 2 (54–59 months children) (*M* = 1.76, *SD* = 1.20, *p* = .017), and Group 1 (48–53 months children) (*M* = 1.44, *SD* = 1.11, *p* = .000).

Eight age groups were formed to determine in which age group(s) the false belief scores of DHH and hearing children differed, and a one-way ANOVA analysis was conducted. Accordingly, it was determined that age made a significant difference in the false belief scores of the groups. As shown in the descriptive statistics presented in Table 2, according to pairwise comparisons, the false belief scores of hearing children in Group 4 were higher than those of the hearing children in Group 1 (*p* = .019) and the DHH children in Group 1 (*p* = .003), Group 2 (*p* = .005), Group 3 (*p* = .005), and Group 4 (*p* = .006). In sum, 5.5 years of age seemed to be the threshold where the false belief scores of DHH and hearing children started to differ in favor of hearing children. There was no significant difference between the false belief scores of other age groups.

The hearing status and mothers’ education level had no interaction effect on the false belief scores. However, the mothers’ education level constituted a significant main effect on false belief scores (see Table 3). When pairwise comparisons were analyzed for all participants, the false belief scores of the children whose mothers were higher education graduates (*M* = 2.46, *SD* = 1.77) were better than those of the children whose mothers were primary education graduates (*M* = 1.39, *SD* = 1.23, *p* = .007).

Similarly, no interaction effect of the hearing status and fathers’ education level on false belief scores was determined. However, the father’s education level had a significant main effect on false belief scores (see Table 3). When the pairwise comparisons were examined, the false belief scores of children whose fathers were higher education graduates (*M* = 2.47, *SD* = 1.70) were better than those of the children whose fathers were primary (*M* = 1.40, *SD* = 1.23, *p* = .000) and secondary education graduates (*M* = 1.61, *SD* = 1.29, *p* = .001).

### Variables specific to hearing loss

Table 4 presents both the descriptive statistics of emotional recognition and false belief scores according to the age at diagnosis, hearing technology, hearing aids, and cochlear implant use of DHH children and the comparison results according to these variables.

**Table 4:** Descriptive statistics and *t*-test analyzes of variables related to hearing loss

**Variables**	**Groups**	**N**	***M***	***SD***	**Min.**	**Max.**	***t***	***p***	${\boldsymbol{\mathrm{\eta}}}^{\mathbf{2}}$
Emotion recognition									
Age at diagnosis	≤3 months	38	4.47	.687	3	5	.045	.964	.01
	>3 months	62	4.47	.620	3	5			
Hearing technology	Hearing aid	32	4.44	.670	3	5	−.345	.731	.07
	Cochlear implant	68	4.49	.635	3	5			
Age at the onset of hearing aid use	≤6 months	37	4.65	.633	3	5	2.17	.032^*^	.44
	>6 months	63	4.37	.630	3	5			
Age at the onset of cochlear implant use	≤36 months	54	4.54	.605	3	5	1.33	.189	.32
	>36 months	14	4.29	.726	3	5			
False belief									
Age at diagnosis	≤3 months	38	1.37	.913	0	3	.069	.945	.17
	>3 months	62	1.36	.977	0	3			
Hearing technology	Hearing aid	32	1.47	.842	0	3	.785	.434	.09
	Cochlear implant	68	1.31	.996	0	3			
Age at the onset of hearing aid use	≤6 months	37	1.49	.961	0	3	1.02	.309	.06
	>6 months	63	1.29	.941	0	3			
Age at the onset of cochlear implant use	≤36 months	54	1.35	.974	0	3	.697	.525	.13
	>36 months	14	1.14	1.10	0	3			

^*^ *p* < .05

As depicted in Table 4, there was a significant difference in the emotion recognition scores of DHH children according to their age at the onset of hearing aid use. Children who had started using hearing aids early had higher emotion recognition scores than those who had started using hearing aids late. There was no significant difference in the emotion recognition scores of DHH children according to the age at diagnosis, hearing technology, and the age at the onset of cochlear implant use. In addition, no significant difference was found in the false belief scores of DHH children according to their age at diagnosis, hearing technology, age at the onset of hearing aid use, and age at the onset of cochlear implant use.

### Variables correlated with DHH children’s emotion recognition and false belief scores

Pearson’s correlation was performed to determine the variables correlated with DHH children’s emotion recognition and false belief scores. The duration of the mothers’ and fathers’ education was included in the analysis by determining the type and length of schooling the parents had completed. The results of the analysis are presented in Table 5.

**Table 5:** Correlations of emotion recognition, false belief, demographic, and audiological variables.

**Variables**	*N*	**2**	**3**	**4**	**5**	**6**	**7**	**8**
1. Age	100	-.12	-.11	.15	.07	.16	.24^*^	.04
2. Duration of mothers’ education (years)	100		.58^*^^*^	-.21^*^	-.26^*^^*^	-.25^*^	.10	.23^*^
3. Duration of fathers’ education (years)	100			-.13	-.22^*^	-.20	.13	.24^*^
4. Age at diagnosis	100				.83^*^^*^	.49^*^^*^	-.05	-.10
5. Age at the onset of hearing aid use	100					.62^*^^*^	-.22^*^	-.20
6. Age at the onset of cochlear implant use	68						-.26^*^	-.23
7. Emotion recognition	100							.60^*^^*^
8. False belief	100							

^*^ *p* < .05, ^*^^*^p < .01.

As displayed in Table 5, there was a significant positive correlation between emotion recognition scores and age, and a significant negative correlation between the age at the onset of hearing aid and cochlear implant use. A significant positive relationship was also found between the false belief scores and the duration of the mothers’ and fathers’ education.

## Discussion

This study examined the emotion recognition and false belief skills of DHH preschool children from a developmental perspective. As a result of the analyses, no significant difference was found between DHH and hearing children’s groups in terms of the emotion recognition scores. In addition to studies consistent with this finding (Ziv et al., 2013), there is also research reporting that hearing children perform better (Dyck et al., 2004; Gray et al., 2007; Huttunen & Ryder, 2012; Most & Michaelis, 2012). The ages of the children in Most and Michaelis’s, and Ziv et al.’s studies are the same as the ages of the participants in the current study. Most and Michaelis concluded that the emotion recognition performances of hearing children (*n* = 14) were better than those of DHH children (*n* = 26), regardless of the visual, auditory, and audio-visual presentations. The higher number of participants in the current study and the fact that the groups were matched in terms of gender and age variables may have led to the homogeneity of the participants. Of course, this alone does not explain the discrepancy between the results, but, in Most and Michaelis’s research, DHH children performed better during audio-visual presentation than other presentations. In the present research, the audio-visual presentation of the test can be considered an important factor in terms of similar performances of DHH and hearing children.

Children at the age of 2 can understand that emotions are different in every person (Wellman, 2014), and at around the same age, they acquire the skills to use emotional expressions and to be aware of others’ emotions (Lewis, 2008). In this respect, children at the age of 4–5 are expected to have acquired the skills of emotion recognition and the use of emotional expressions. The DHH children in this study were 4–5 years old, and although they may have experienced some language delays due to hearing loss, they might be expected to perform the skills that are normally acquired at age 2 by the time they are 4–5 years old.

False belief understanding is known to develop at ~4 years of age. For a specific analysis, the false belief performances of DHH and hearing children divided into four age groups were analyzed separately within the groups. It was noted that the performances of DHH children were similar whereas the performances of hearing children showed a significant age-related difference. The fact that there was no age-related difference in the false belief performances of DHH children may be attributed to the narrow age range of the participants. Indeed, the studies performed with DHH children have concluded that there is an age-related change in false belief performance and there is a relationship between age and ToM (Fujino et al., 2017; Smogorzewska & Osterhaus, 2021; Walker et al., 2017).

Several studies on false belief understanding reported that hearing children performed better than DHH children (Netten et al., 2017; Peterson et al., 2016; Pluta et al., 2021; Ziv et al., 2013). Pluta et al. compared the false belief performances of implant users and hearing children at the age of 3–8 years and reported that DHH children performed poorer than their peers in terms of false belief understanding by age 6. However, in the present study, the DHH and hearing children performed similarly in false belief understanding until the age of 5.5. Ziv et al. compared the false belief skills of 5–7-year-old DHH and hearing children and reported that DHH children using oral language performed similarly to their hearing peers in false belief understanding. In contrast to their findings, the present study concluded that DHH children had similar performances to those of their hearing peers at younger ages. In this study, the children were divided into groups based on their age into 6-month periods, and the false belief scores in each age group were analyzed. Ziv et al., on the other hand, analyzed false belief scores without grouping children according to age. In other words, they did not examine whether false belief scores changed with age. Moreover, their participants were older than the participants in the present study. Considering these findings, the DHH children in the present study may require a few more years to succeed in false belief tasks.

Lack of language and social experiences and exposure to less language-rich environments at young ages can negatively affect language development and the rate of ToM development (Peterson et al., 2012; Ziv et al., 2013). This could explain the difference in the false belief performances of DHH and hearing children at the age of 5.5 in the present study. From another perspective, the duration of parental education was longer for the hearing children than for the DHH children. This difference in the duration of education may have been reflected in the differences observed in children’s false belief performances. However, the important finding in this study is that the false belief performances of the groups were similar between the ages of 4 and 5.5. Due to the factors mentioned at the beginning of this paragraph, the slow development of DHH children is a predicted result. In this framework, the negative experiences at an early age could turn into disadvantages in the long term. From a developmental point of view, there is a 5.5-year period with no difference between groups. This does not mean that DHH children do not have any disadvantages until this age. It means that potential disadvantages may start to emerge during this period due to the nature of false belief development. In fact, this suggests the need to observe the trajectory of false belief development beyond this age. Because development is a holistic process, false belief understanding should be regarded as an accumulation of children’s experiences since birth. These experiences may also be influenced by parents’ education level, who interact with children the most. In this study, a significant positive correlation was found between the false belief scores of DHH children and the duration of parents’ education (duration of mothers’ education, *r* = .23, *p* < .05; duration of fathers’ education, *r* = .24, *p* < .05). In addition, although there was no interaction effect with hearing status, parental education level was found to have a main effect on false belief scores. When these findings were considered together, the indirect contribution of parents’ high level of education on child development may have enabled the DHH children to perform similarly to their peers until the age of 5.5.

Unlike the present study, parents’ education level was not taken as a separate variable in previous research but was evaluated within the socioeconomic level. Although these studies emphasized that socioeconomic level was an important factor in the development of ToM (Hughes et al., 2005; Shatz et al., 2003), Devine and Hughes (2018), in their meta-analysis, concluded that the socioeconomic level was not an influential variable in ToM research in recent years. Given that the findings are not conclusive, it may be plausible to regard parental education level as a separate variable as in the present study to improve the clarity of results.

In a study conducted with DHH children in preschool period, it was reported that there was a significant positive correlation between the duration of mothers’ education and false belief and ToM scores (Pears & Moses, 2003). The findings suggested that the duration of the parents’ education was a remarkable factor in children’s false belief understanding.

The positive influence of parents’ higher education level on the false belief performances of DHH children may have various dimensions. Factors such as an increase in the socioeconomic status associated with the higher education level, the parents’ awareness of decisions made about their child, and their participation in family education may contribute to this positive influence. Parents’ awareness and higher socioeconomic status can facilitate access to resources, allowing for early diagnosis and intervention for the DHH child, including early fitting of hearing aids and early initiation of education. The negative correlation between duration of mothers’ education and age at diagnosis (*r* = −.21, *p* < .05), age at the onset of hearing aid use (*r* = −.26, *p* < .01), and age at the onset of cochlear implant use (*r* = −.25, *p* = .05) can be seen as positive consequences of mothers’ education level on their children’s lives.

Analysis of the emotion recognition and false belief performances of DHH children in terms of variables related to hearing loss showed that hearing technology used by children led to no difference in their emotion recognition and false belief scores. It may be related to the fact that the majority of children using implants (*n* = 68) had early implants (*n* = 54) and the majority of children using hearing aids (*n* = 32) had moderate hearing loss (*n* = 26). In line with the findings of the present study, the type of hearing technology used in previous studies did not make a difference in false belief performances (Moeller & Schick, 2006; O'Reilly et al., 2014; Peterson, 2004).

The emotion recognition scores of DHH children did not differ according to when they started cochlear implant use, but the younger the children were when the implant was placed the higher emotion recognition scores they got. The reason why the influence of the implant was not observed may be related to the fact that emotion recognition develops at an early age and can be clearly understood from facial expressions. However, the significant negative correlation between the age at the onset of implant use and the emotion recognition scores can be interpreted as the benefit of the implant. That the children with implants in present study wore hearing aids before the implantation may also have contributed to the acquisition of emotion recognition skills. This is because the age at the onset of hearing aid use is a remarkable factor in emotion recognition performance, and there is a negative relationship between the age at the onset of hearing aid use and emotion recognition scores. Considering the positive correlation between the age at the onset of cochlear implant use and the age at the onset of hearing aid use (*r* = .62, *p* < .01), it is possible to conclude that the use of any hearing technology has a positive contribution to the acquisition of emotion recognition skills.

No significant relationship was found between how children perform in false belief understanding and the age at the onset of cochlear implant use. However, Yu et al. (2021) reported that cochlear implant use predicted the performance in false belief understanding with language as a mediating variable. The mean age at the onset of cochlear implant use for the children in Yu et al.’s study was younger than that of the children in this study, and their study included children older than 72 months. Therefore, it is feasible to state that the children in Yu et al.’s study have used the implant for a longer duration than the children in the present study. This factor may account for the differences between the findings of these two studies.

### Limitations of the research and suggestions

The lack of measurements based on parent or teacher reports/statements can be considered a limitation. The evaluation of emotion recognition and false belief performance based on adult reports could have provided support for task-based evaluation.

The most important recommendation based on the results of this research is to examine age-related development by evaluating false belief understanding of DHH children in a wider age range. In addition, other components of ToM such as diverse desires, diverse beliefs, knowledge access, hidden emotions, faux pas, and irony may also be investigated in further research endeavors.

Research can also be carried out on the relationship between false belief, emotion recognition, and language skills. The relationship among these skills and social competence may be examined. Studies comparing the false belief and emotion recognition skills of DHH children who have hearing parents to DHH children who have DHH parents may be conducted. ToM studies can be designed in which sign language is included as a variable.

Finally, relevant suggestions can be helpful to the practitioners regarding the significant positive relationship between parental education level and false belief scores, which are remarkable findings in the present study. Based on this relationship, data can be collected on how parents with high education level interact with their DHH children and what they do for the educational, social, and developmental progress of their children. This information can be integrated into a family education program. In addition, interaction groups can be formed with parents who have a high level of education and those with a low level of education to share their experiences.

## Data Availability

The data used in this research will be shared if the authors opt to do so.

## Notes

This article is based on the master’s thesis completed by first author under the supervision of second author.
