# Correction to: Emotion recognition and false belief in deaf or hard-of-hearing preschool children

**DOI:** 10.1093/deafed/enad047

**Published:** 2023-10-19

This is a correction to: Emrah Akkaya, Murat Dogan, Emotion recognition and false belief in deaf or hard-of-hearing preschool children, *The Journal of Deaf Studies and Deaf Education*, 2023; enad044, https://doi.org/10.1093/deafed/enad044

In the originally published version of this manuscript, in section Method there was an error in section subtitled The Theory of Mind Task Battery for Children-Turkish Version. The first part of the 2nd sentence should read: “There is a total of 26 items in the test, [...]” instead of: “There is a total of 25 items in the test, [...]”. There was an error under section Data analysis, part of third sentence should read: “[...]age at diagnosis of DHH children,[...]”. instead of: “[..]diagnostic age of DHH children,[..]”. In Table 3., “mother's” and “father's” should read: “mothers”' and “fathers”' throughout. In References, the erroneous insertion “& ve” was emended to “&” in the author lists of three references.

These errors have been corrected in the article.

